# Delayed Diagnosis of Osteonecrosis of the Jaw (ONJ) Associated with Bevacizumab Therapy in Colorectal Cancer Patients: Report of Two Cases

**DOI:** 10.3390/dj4040039

**Published:** 2016-10-29

**Authors:** Francesco Erovigni, Alessio Gambino, Marco Cabras, Antonella Fasciolo, Silvio Diego Bianchi, Elisa Bellini, Vittorio Fusco

**Affiliations:** 1 Oral Surgery Department, Dental School, AOUP Città della Salute e della Scienza di Torino, Torino 10100, Italy; cabrasmarco300@gmail.com; 2 Maxillofacial Unit, Alessandria Hospital, Alessandria 15121, Italy; AFasciolo@ospedale.al.it; 3 Radiology Department, Dental School, AOUP Città della Salute e della Scienza di Torino, Torino 10100, Italy; silviodiego.bianchi@unito.it; 4 Oncology Unit, Molinette Hospital, AOUP Città della Salute e della Scienza di Torino, Torino 10100, Italy; ebellini@cittadellasalute.to.it; 5 Oncology Unit, Alessandria Hospital, Alessandria 15121, Italy; 6 Centro di Documentazione Osteonecrosi, Alessandria 15121, Italy

**Keywords:** colorectal cancer, bevacizumab, osteonecrosis of jaw, ONJ, MRONJ, bisphosphonate

## Abstract

Medication-induced Osteonecrosis of the Jaw (MRONJ) has been reported not only after use of antiresorptive agents (bisphosphonates and denosumab), but also in cancer patients receiving antiangiogenic agents, alone or combined with antiresorptive drugs. We report two cases of MRONJ observed in colorectal cancer patients after bevacizumab therapy only. MRONJ was diagnosed, respectively, two and seven months after a tooth extraction; both the patients had received two courses of bevacizumab infusions (for a total of 29 and 10 administrations, respectively). We discuss if tooth extraction during or after antiangiogenic therapy could be a potential trigger of MRONJ, but also if an underlying bone disease not evident before oral surgery might be a possible cause. A careful drug history has to be registered by dental specialists in cancer patients before oral surgery and adequate imaging might be obtained to avoid a delayed diagnosis.

## 1. Introduction

Osteonecrosis of the Jaw (ONJ) has been reported in patients treated with bisphosphonates since 2003 [1]. Bisphosphonate-Related Osteonecrosis of the Jaw (BRONJ) was defined by AAOMS (American Association of Oral Maxillofacial Surgeons) in 2007 as the presence of exposed, necrotic bone in the maxillofacial region that has persisted for more than eight weeks in patients with current or previous treatment with bisphosphonates, and no history of head and neck radiation to the jaws [2]; however, the occurrence of cases without bone exposure questioned that definition [3,4,5].

Recently the BRONJ definition has been amended by AAOMS to Medication-Related Osteonecrosis of the Jaw (MRONJ) to integrate the growing number of osteonecrosis cases involving the maxilla and mandible associated with another antiresorptive drug (denosumab) and targeted therapies, including antiangiogenic agents [6]; the disease definition was also slightly changed to include “bone that can be probed through an intraoral or extraoral fistula in the maxillofacial region” [6].

Some anticancer antiangiogenic agents inhibit Vascular Endothelial Growth Factor (VEGF) and may increase the risk of osteonecrosis of the jaw [7,8].

Bevacizumab is a recombinant humanized monoclonal antibody and it blocks angiogenesis by binding to Vascular Endothelial Growth Factor Receptor (VEGFR); indications for bevacizumab include metastatic cancer of breast, lung, colon, rectum, kidney, ovarian cancer, and glioblastoma. Bevacizumab side effects include thromboembolic episodes, hypertension, hemorrhage, gastrointestinal perforation, and wound-healing complications.

ONJ occurrence related to bevacizumab treatment was first reported by Estilo et al. in 2008 [9]. Since then, other cases have been described in cancer patients [10,11,12,13,14,15,16,17,18], including colorectal cancer patients [19,20].

Two unusual cases of bevacizumab-related MRONJ in patients who received chemotherapy associated to bevacizumab without any bisphosphonate therapy are reported here.

## 2. Case Report

### 2.1. Case Report 1

**Figure 1:**
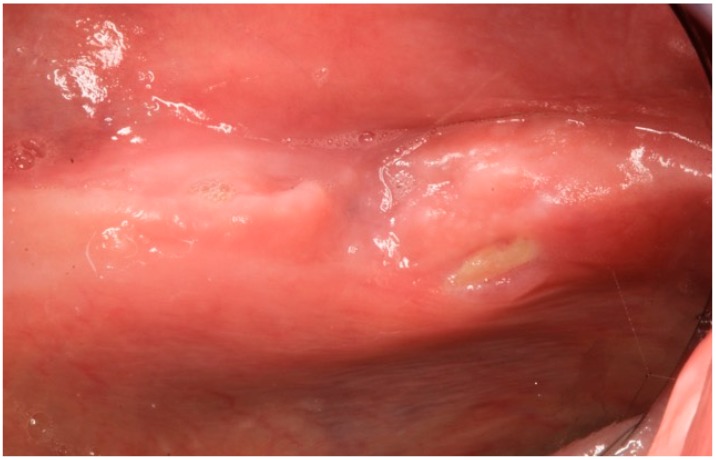
Case 1: Intraoral aspect of the bone exposition on left the mylohyoid line.

A 79-year-old male patient was referred on December 2015 to the Turin Oral Health Department (Dental School) for a baseline exam before starting therapy with bisphosphonates; clinical examination revealed an already present bone exposition approximately 3 mm × 1 mm in the left posterior mandible, in correspondence of the mylohyoid line, asymptomatic (Figure 1).

The history from the patient included colon adenocarcinoma that was diagnosed and treated surgically on October 2008 (stage pT2 pN1 G3). Subsequently the patient underwent adjuvant chemotherapy with the XELOX scheme (capecitabine and oxaliplatin).

He also suffered from hypertension and prostatic hyperplasia, and had been submitted for coronary angioplasty due to cardiac ischemia in 2007.

On September 2010, the patient underwent surgery of lung nodules (pathology revealed a lung hamartoma and some metastases of carcinoma of colorectal origin). From November 2010 to April 2011, he received postoperative chemotherapy with the FOLFIRI scheme (irinotecan, fluorouracil, folinic acid).

After the lung progression of the disease, from January 2012 to March 2012 he underwent FOLFOX chemotherapy (oxaliplatin, fluouracil, folinic acid) which was stopped for an allergic reaction to oxaliplatin. In April 2012, he started chemotherapy with irinotecan together with bevacizumab (7.5 mg/kg every 14 days) for seven cycles (until June 2012). After a drug holiday, on October 2013 the patient restarted chemotherapy (without bevacizumab) for five other cycles. On March 2014, he underwent stereotactic radiation therapy of two residual lung nodules.

Due to a new lung progression, on October 2014 he started irinotecan and bevacizumab again (same doses) for 12 cycles, until April 2015. He continued bevacizumab only (maintenance therapy) until October 2015 for 10 courses, until reaching a total of 22 bevacizumab administrations in this second course.

On October 2015, bone metastases were diagnosed and he started therapy with mitomycin and fluorouracil.

On 1 October 2015, the patient had undergone tooth extraction of the two mandible canines and of the second left mandible molar due to excessive mobility at a private dentist office. Tooth extractions were conducted under antibiotic coverage (amoxicilline plus clavulanic acid 1 g every 12 h for six days).

**Figure 2:**
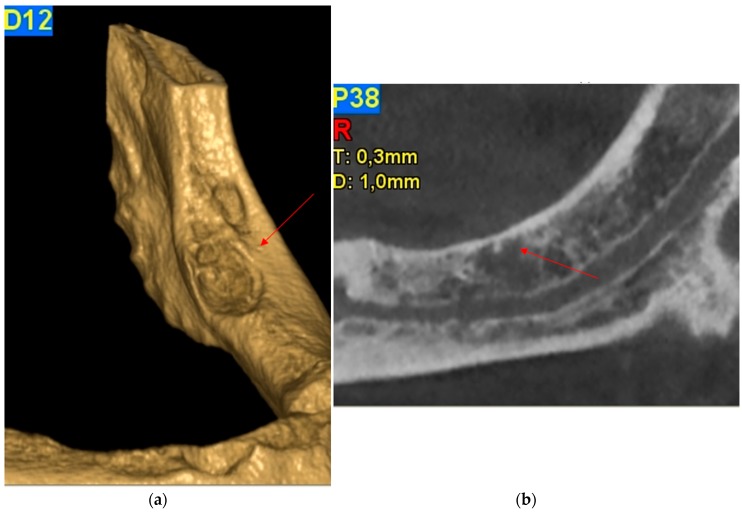

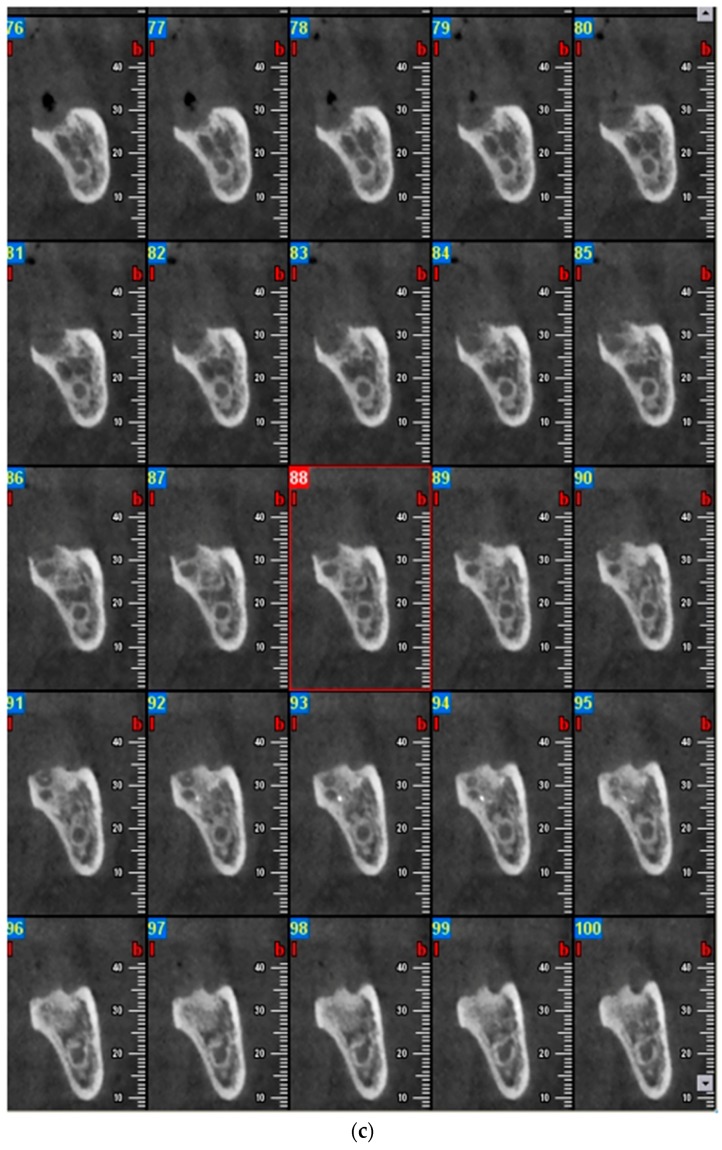
Case 1: Cone-beam computer tomography images: (**a**) 3D reconstruction; (**b**) Panorex; (**c**) CT scan images of the bone exposition.

On December 2015, the patient was referred to the Turin Oral Health Department (Dental School) for a baseline evaluation in order to start intravenous bisphosphonate therapy, after diagnosis of bone metastases. At the time of the visit the patient was completely edentulous, and he was a wearer of two dentures. As written before, intraoral examination showed a bone exposition approximately 3 mm × 1 mm in the left posterior mandible, in correspondence of the mylohyoid line, asymptomatic (Figure 1). Trauma from the dentures was excluded. The surrounding soft tissue appeared ulcerated and necrotic, with no evidence of infection, so a cone-beam computer tomography (CBCT) was requested in order to investigate the real dimensions of the necrosis and to exclude metastatic lesions. CBCT revealed a minor cortical sclerotic bone lesion and a residual of the post-extraction socket without images of bone sequestra (Figure 2a–c).

**Figure 3:**
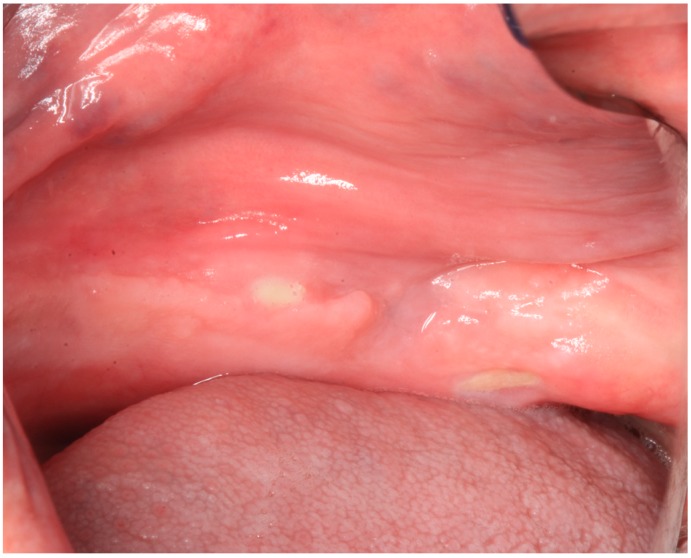
Case 1: Oral aspect of the bone exposition with the oral fistula.

The patient underwent six administrations of low level laser therapy (LLLT), twice a week, and then he was re-evaluated. At the end of January 2016, the patient underwent a contrast-enhanced computer tomography (CT) scan, which excluded definitively the hypothesis of a metastatic lesion. At that date, the lesion was still asymptomatic, but an oral fistula appeared in the second left molar region (Figure 3). The diagnosis of MRONJ was confirmed.

A new antibiotic therapy was prescribed (amoxicilline plus clavulanic acid 1 g every 8 h for six days and metronidazole 500 mg every 8 h for six days) in combination with a chlorhexidine 0.2% oral rinse. After a week the patient expelled a bone sequestrum approximately 4 mm × 2 mm large as well as referred mucosal healing at the bone exposure site and disappearance of the oral fistula.

**Figure 4:**
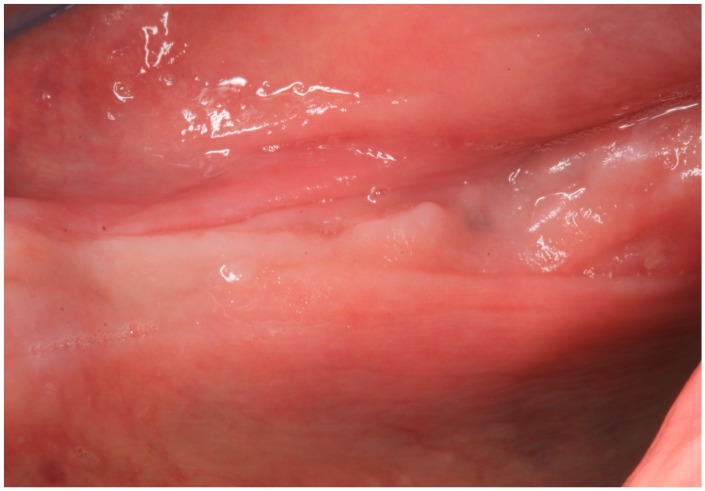
Case 1: Resolution of the bone exposition.

On 3 March 2016, the patient was re-evaluated and at intraoral examination both the bone exposure and the oral fistula were completely healed (Figure 4).

At six months from the event, there were no recurrences. Further follow-up visits are planned.

### 2.2. Case Report 2

A 60-year-old man was visited at the Alessandria Hospital Oncology Unit on August 2011 for a routine visit before a chemotherapy administration (FOLFOX scheme and bevacizumab), complaining of left mandible pain (VAS: visual analogue scale, score 6) and an oral mucosal break; the oral examination revealed a lingual-side bone exposure, 1 cm in diameter (no photo available). Four weeks before, he had started treatment with chemotherapy associated with bevacizumab for the second time.

His cancer history had begun on February 2010 with the diagnosis of rectal cancer and lung metastases. He underwent treatment with pelvic radiotherapy and simultaneous chemotherapy (oxaliplatin and fluorouracil in a continuous infusion) and then rectal surgery (on July 2010); additional chemotherapy (FOLFIRI) was administered in August 2010. Some pulmonary metastases were surgically removed on November 2010 but residual disease was still present in a chest postoperative CT scan.

He received chemotherapy (FOLFIRI scheme) and bevacizumab (5 mg/kg day 1 every 14 days) between December 2010 and March 2011 (eight cycles over four months). Due to pulmonary progression of the disease, he underwent a short treatment with an experimental agent in another cancer center, suspended on July 2011 due to the diagnosis of brain metastases, treated with radiotherapy. He then started new chemotherapy (FOLFOX scheme) with bevacizumab again.

His dental history revealed that he had been submitted for left mandible third molar (3.8) extraction at a private dental office on January 2011 (during the first bevacizumab treatment) without notifying the treating oncologist; the patient complained about incomplete and delayed socket closure after extraction.

**Figure 5:**
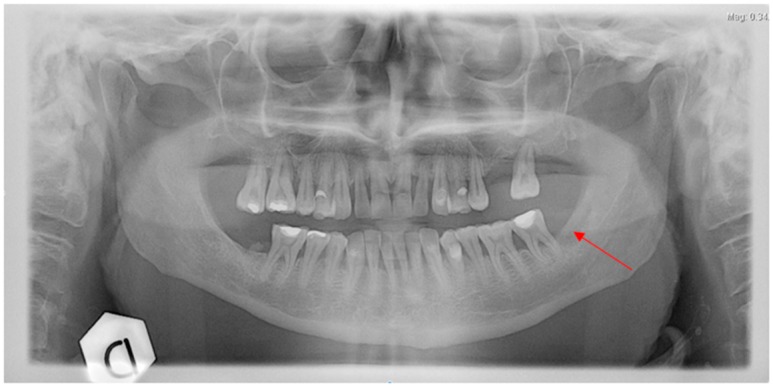
Case 2: Rx orthopatogram of the post-extractive socket.

**Figure 6:**
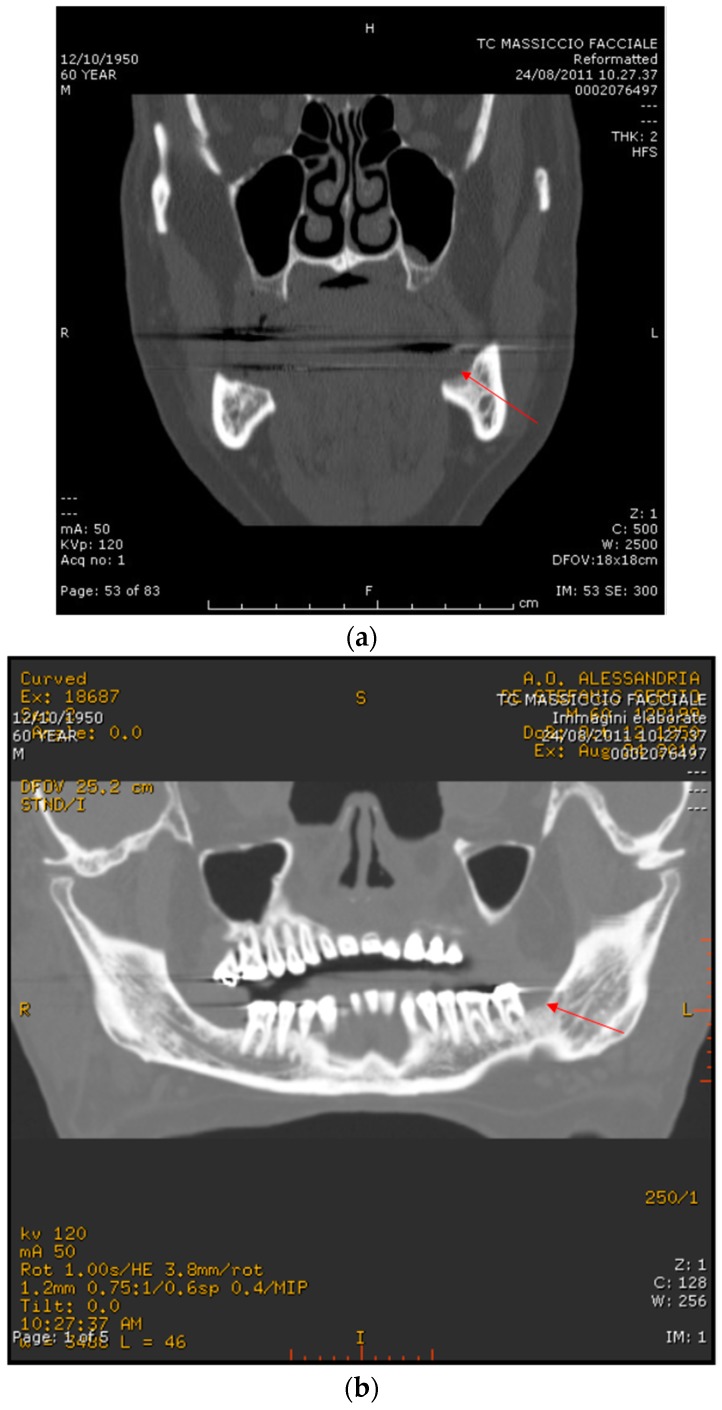

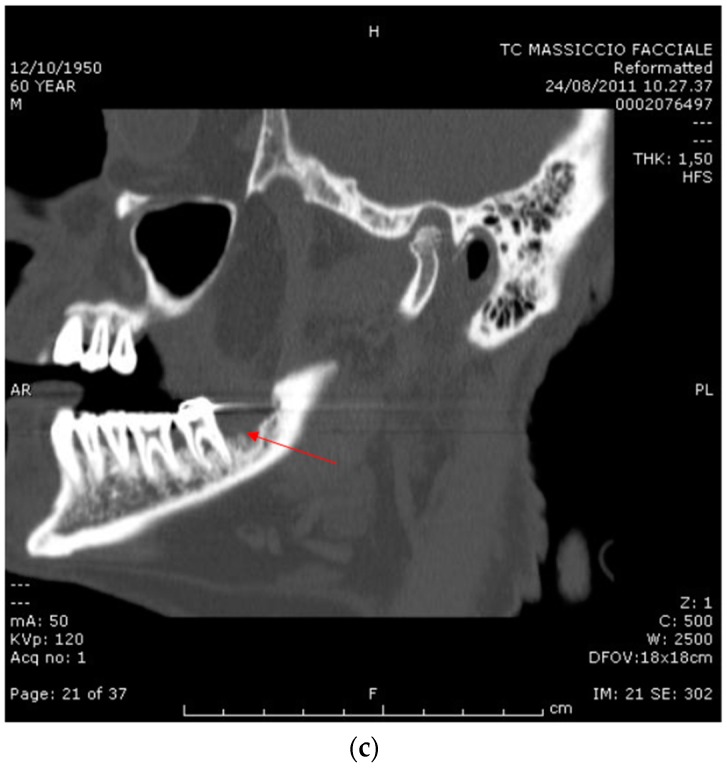
Case 2: CT scan images of the mandible post-extractive socket. (**a**) Coronal formatting; (**b**) Panorex; (**c**) Sagittal formatting that shows the bone sequestrum.

On August 2011, at an oncology unit visit shortly after the start of the second course of bevacizumab therapy (two administrations), a painful mandible bone exposure was noted and the patient was referred to the Alessandria Hospital ONJ multidisciplinary team; antibiotics (amoxicilline plus clavulanic acid 1 g every 8–12 h and metronidazole 500 mg every 12 h) in combination with a chlorhexidine 0.12% oral rinse were prescribed, with immediate pain reduction. Rx ortopantomography showed no signs of osteolysis but just the profile of the residual socket at the extraction site (Figure 5). A CT scan without contrast showed a mandible bone cortical lesion in the previous tooth extraction site, 7 mm × 4 mm in size (Figure 6a–c). Spontaneous expulsion of a bone fragment was referred by the patient two weeks later. The patient was not further visited by the ONJ multidisciplinary team due to death shortly after an episode of pulmonary embolism on October 2011.

## 3. Discussion

### 3.1. Origin of ONJ

Development of drug-related ONJ, now known as MRONJ, has been reported to occur spontaneously or be facilitated by tooth extractions, dental procedures, poor oral hygiene, and infections [1]. Pathogenesis of bisphosphonate-related ONJ (BRONJ) is believed to be multifactorial, involving altered bone turnover, infection, altered immune system, but also inhibition of angiogenesis by bisphosphonates [21,22,23,24,25,26,27,28]. Furthermore, BRONJ more frequently affects the mandible than the upper jaw; this effect could be attributed to the relatively poor vascularization of the mandible compared to the maxilla.

Studies show that antiangiogenic agents can increase the risk for jaw osteonecrosis in cancer patients, particularly when combined with bisphosphonates [8,29], but also when administered in patients not receiving antiresorptive agents. This is well known for bevacizumab [9,10] and sunitinib [30], but also other drugs with antiangiogenic effects [8,31,32,33].

### 3.2. ONJ after Bevacizumab Therapy

In the current report, we describe two cases of osteonecrosis in colorectal cancer patients treated with bevacizumab without any prior bisphosphonate exposure.

Osteonecrosis of the lower jaw due to bevacizumab therapy has been described in the literature, with the first two cases being described by Estilo in [9]. Bevacizumab is a recombinant humanized IgG monoclonal antibody that binds Vascular Endothelial Growth Factor (VEGF) prior to its linkage to the cell receptors, and neutralizes its activity. It is involved in the treatment of selected advanced colorectal, lung, breast, renal and central nervous system tumors and plays a developing role in the management of ovarian and cervix cancers.

Angiogenesis is involved in tumor development, growth and metastasis; consequently, the intracellular signaling pathways related to VEGF represent a potential target for anticancer therapy [34,35].

Upregulation of VEGF-mediated angiogenesis is also a crucial step of the inflammatory response in wound healing [36]. Bevacizumab inhibits tissue repair by preventing new blood vessel formation and the migration of inflammatory cells and essential nutrients to the diseased tissue [37].

It has also been shown that VEGF may regulate osteoclast differentiation and directly stimulates osteoclastic bone resorption by enhancing the survival of mature rabbit osteoclasts [38]. Consequently, in patients receiving bevacizumab osteoclasts, inactivation and inhibition of neo-angiogenesis could hamper the normal bone repair mechanism, leading to the accumulation of avascular and non-viable bone.

Interestingly, the antiangiogenic effects of bevacizumab are dose-dependent and time-dependent [39]. This could imply that angiogenesis, bone remodeling and healing processes are able to restart after drug cessation. The estimated half-life of bevacizumab is approximately 20 days (range 11–50 days).

This observation is in line with the observation of bevacizumab-related ONJ as a self-limiting disease [14].

Furthermore, recent studies show that periodontal disease preceding dental extractions in patients treated with antiresorptives alone or in combination with targeted therapy may represent developing osteonecrosis [40].

### 3.3. Our Two Cases

The two cases herein described question the possible mechanism of bevacizumab-induced ONJ.

In the first case described above, the patient stopped bevacizumab on July 2015 after 22 cycles (started on October 2014) and underwent the dental extractions on October 2015 (three months after). One could see the tooth extraction as a “trigger” of ONJ, but, alternatively, the reason for the extraction, i.e., excessive tooth mobility, could be interpreted as a sign of an already underlying bone disease. An exposed bone area (compatible with a diagnosis of MRONJ) was firstly registered on December 2015; according to the classical BRONJ and MRONJ definition [2,6,41], the patient should have been only observed for eight weeks to reach a definitive diagnosis. We generally do not agree with this “wait and see” attitude and the patient underwent medical and laser therapy. In March 2016, we observed resolution of the mucosal break after expulsion of bone sequestra (another event, together with bone exposure and CT images, confirming a clear diagnosis of ONJ even in the absence of the eight-week observation requested by AAOMS, in our opinion).

The combined inhibitory effect of bevacizumab on mucosal healing and on bone remodeling may be responsible for the start of the osteonecrotic process after the dental extraction, if one considers the surgery as the trigger of ONJ. The risk of wound dehiscence in patients treated with bevacizumab is considered inversely proportional to the interval from bevacizumab injection to surgical treatment; however, complications in wound healing can occur until 12 months after drug exposure [42].

Conversely, one could consider the hypothesis of an underlying bone alteration already present at the extraction time, with the excessive tooth mobility as a precocious sign of “hidden” ONJ: this theory could have been explored before extraction only with a CT scan, which is able to detect bone sclerosis and other bone alterations linked to drug exposure (i.e., bisphosponate, but even other agents) in patients without bone exposure [43,44]. Also bone scintigraphy (a staging and diagnostic tool routinely prescribed in some advanced cancer patients) is able to show tracer uptake at an ONJ site, at ONJ diagnosis time or even several months before [45], although with some specificity problems. Clearly, the prescription of a CT scan in the absence of frank bone exposure should be guided by suspicious symptoms or signs (pain, infections, tooth mobility, spontaneous tooth loss, etc.) in alerted physicians or dentists. In this case, the diagnosis of ONJ (based on clinical suspicions and on imaging studies, and, above all, according to a less restricted ONJ definition) might be more precocious.

In the second case, again the ONJ diagnosis was not made as early as possible. It appeared with a painful mandible bone exposure observed shortly after the start of a second course of bevacizumab treatment, but the cause was probably due to the first course of bevacizumab treatment administered some months before, during which the patient underwent a tooth extraction with a delayed and incomplete socket closure. As, in this case, we do not exactly know the reason that caused an un-alerted practice dentist to do a tooth extraction (two months after the bevacizumab treatment start), it is more probable that the extraction need is attributable to a pre-existing oral disease together with the bevacizumab effect. Also, in this case, a higher communication level between the dentist and oncologist and, above all, a full awareness of the bevacizumab-related ONJ risk might have led to avoiding the tooth extraction (if possible) or at least conducting an imaging study (CT scan) to reach an earlier diagnosis of ONJ disease in the patient history.

Another issue is for what duration is the bevacizumab treatment needed to induce ONJ. There is a considerable variation in the timing of the onset of ONJ, which occurred at any time between one to 12 months after the start of bevacizumab in the reported ONJ cases not receiving bisphosphonates [9,10,11,12,13,14,16,17,18,19,20], with the significant exception of one case of ONJ diagnosed 10 months after the last bevacizumab administration, and about two years after the start of treatment [15].

However, one has to consider that the measure of time from the first bevacizumab administration to ONJ diagnosis (or suspect) can largely change according to the clinician’s awareness and to the single case’s disease process characteristics. Furthermore, in our two cases this evaluation is made even more difficult as the two patients received bevacizumab in two different courses along with their anticancer treatment history: until ONJ registration by multidisciplinary team members, the time interval from the antiangiogenic start was, respectively, 44 and 14 months in the first patient, and eight months and one only month in the second case. Anyway, our two cases are representative of a large population of metastatic colorectal patients nowadays reaching prolonged survival after new treatments, often receiving more and more lines of treatment [8].

### 3.4. Frequency of Bevacizumab-Related ONJ: the Possible Underestimation and the Next Future

On November 2010, after the first literature papers and congress reports, the European drug regulatory agency launched an alert about ONJ risk [46]; a “dear doctor” letter reported data from the bevacizumab manufacturer about 55 bevacizumab-related cases of ONJ among approximately 800,000 patients undergoing treatment [46]. The real frequency of bevacizumab-related ONJ cases is unknown, due to a lack of reporting to surveillance systems [8] but also due to reduced clinicians’ awareness, as our two cases show.

Furthermore, another reason for the underestimation is probably a too-restricted traditional ONJ definition, excluding cases without bone exposure [47].

In recent years, colorectal patients with metastatic disease have reached prolonged median survivals and receive several lines of treatment, also including antiangiogenic and other targeted therapies. Consequently, the ONJ risk increases along with prolonged and repeated treatments (as shown in our two cases), with therapy including several agents [32,33,42], and with longer observation times. So we have to expect more and more similar ONJ cases will be observed in real life settings.

### 3.5. Conclusive Remarks

Clinicians and dentists need to be aware of MRONJ as a potential side effect of bevacizumab treatment, occurring often after tooth extractions or oral surgery, but sometimes independently of them.

Bevacizumab therapy can affect the planning of surgery as it might impact the wound-healing process. The interval from the discontinuation of bevacizumab to surgery that should be respected to avoid surgical complications has not been clearly defined. It has been advised that bevacizumab should be stopped at least five to eight weeks before a surgical intervention and restarted four weeks later or when the wound is fully healed [48].

If patients undergoing antiangiogenic treatment (bevacizumab, sunitinib, etc.) without antiresorptive agents should undergo preventive dentistry measures before starting treatment (as is recommended for patients receiving antiresorptive drugs) has to be explored.

In the case of the increasing number of bevacizumab-associated ONJ cases, special dental management (jaw X-ray, measures for optimal dental health and good oral hygiene) could become standard before patients start bevacizumab therapy.

## References

[1] Ruggiero S.L. (2009). Bisphosphonate-related osteonecrosis of the jaw (BRONJ): Initial discovery and subsequent development. J. Oral Maxillofac. Surg..

[2] Advisory Task Force on Bisphosphonate-Related Ostenonecrosis of the Jaws, American Association of Oral and Maxillofacial Surgeons (2007). American association of oral and maxillofacial Surgeons position paper on bisphosphonate-related osteonecrosis of the jaws. J. Oral Maxillofac. Surg..

[3] Colella G., Campisi G., Fusco V. (2009). American Association of Oral and Maxillofacial Surgeons position paper: Bisphosphonate-Related Osteonecrosis of the Jaws-2009 update: The need to refine the BRONJ definition. J. Oral Maxillofac. Surg..

[4] Bedogni A., Fusco V., Agrillo A., Campisi G. (2012). Learning from experience. Proposal of a refined definition and staging system for bisphosphonate-related osteonecrosis of the jaw (BRONJ). Oral Dis..

[5] Otto S., Marx R.E., Tröltzsch M., Ristow O., Ziebart T., Al-Nawas B., Groetz K.A., Ehrenfeld M., Mercadante V., Porter S. (2015). Comments on “diagnosis and management of osteonecrosis of the jaw: A systematic review and international consensus”. J. Bone Miner. Res..

[6] Ruggiero S.L., Dodson T.B., Fantasia J., Goodday R., Aghaloo T., Mehrotra B., O’Ryan F. (2014). American Association of Oral and Maxillofacial Surgeons. American Association of Oral and Maxillofacial Surgeons position paper on medication-related osteonecrosis of the jaw—2014 update. J. Oral Maxillofac. Surg..

[7] Troeltzsch M., Woodlock T., Kriegelstein S. (2012). Physiology and Pharmacology of Nonbisphosphonate Drugs Implicated in Osteonecrosis of the Jaw. J. Can. Dent. Assoc..

[8] Fusco V., Santini D., Armento G., Tonini G., Campisi G. (2016). Osteonecrosis of jaw beyond antiresorptive (bone-targeted) agents: New horizons in oncology. Expert Opin. Drug Saf..

[9] Estilo C.L., Fornier M., Farooki A., Carlson D., Bohle G., Huryn J.M. (2008). Osteonecrosis of the jaw related to bevacizumab. J. Clin. Oncol..

[10] Greuter S., Schmid F., Ruhstaller T., Thuerlimann B. (2008). Bevacizumab-associated osteonecrosis of the jaw. Ann. Oncol..

[11] Serra E., Paolantonio M., Spoto G., Mastrangelo F., Tetè S., Dolci M. (2009). Bevacizumab-related osteonecrosis of the jaw. Int. J. Immunopathol. Pharmacol..

[12] Guarneri V., Miles D., Robert N., Diéras V., Glaspy J., Smith I., Thomssen C., Biganzoli L., Taran T., Conte P. (2010). Bevacizumab and osteonecrosis of the jaw: Incidence and association with bisphosphonate therapy in three large prospective trials in advanced breast cancer. Breast Cancer Res. Treat..

[13] Agostino N.M., Gingrich R., Drabick J.J. (2010). Bevacizumab demonstrates prolonged disease stabilization in patients with heavily pretreated metastatic renal cell carcinoma: A case series and review of the literature. Adv. Urol..

[14] Bettini G., Blandamura S., Saia G., Bedogni A. (2012). Bevacizumab-related osteonecrosis of the mandible is a self-limiting disease process. BMJ Case Rep..

[15] Brunamonti Binello P., Bandelloni R., Labanca M., Buffoli B., Rezzani R., Rodella L.F. (2012). Osteonecrosis of the jaws and bevacizumab therapy: A case report. Int. J. Immunopathol. Pharmacol..

[16] Pakosch D., Papadimas D., Munding J., Kawa D., Kriwalsky M.S. (2013). Osteonecrosis of the mandible due to anti-angiogenic agent, bevacizumab. Oral Maxillofac. Surg..

[17] Santos-Silva A.R., Belizário Rosa G.A., Castro Júnior G.D., Dias R.B., Prado Ribeiro A.C., Brandão T.B. (2013). Osteonecrosis of the mandible associated with bevacizumab therapy. Oral Surg. Oral Med. Oral Pathol. Oral Radiol..

[18] Tzermpos F., Ismail A., Pavli M., Tosios K.I. (2016). Osteonecrosis of the mandible in a patient with lung adenocarcinoma undergoing anti-angiogenic therapy with bevacizumab. Oral Surg..

[19] Dişel U., Beşen A.A., Özyılkan Ö., Er E., Canpolat T. (2012). A case report of bevacizumab-related osteonecrosis of the jaw: Old problem, new culprit. Oral Oncol..

[20] Sato M., Ono F., Yamamura A., Onochi S. (2013). A case of osteonecrosis of the jaw during treatment by bevacizumab for sigmoid colon cancer. Nihon Shokakibyo Gakkai Zasshi.

[21] Santini D., Galluzzo S., Vincenzi B., Schiavon G., Fratto E., Pantano F., Tonini G. (2007). New developments of aminobisphosphonates: The double face of Janus. Ann. Oncol..

[22] Allen M.R., Burr D.B. (2009). The pathogenesis of bisphosphonate-related osteonecrosis of the jaw: So many hypotheses, so few data. J. Oral Maxillofac. Surg..

[23] Vincenzi B., Napolitano A., Zoccoli A., Iuliani M., Pantano F., Papapietro N., Denaro V., Santini D., Tonini G. (2012). Serum VEGF levels as predictive marker of bisphosphonate-related osteonecrosis of the jaw. J. Hematol. Oncol..

[24] Arduino P.G., Menegatti E., Scoletta M., Battaglio C., Mozzati M., Chiecchio A., Berardi D., Vandone A.M., Donadio M., Gandolfo S. (2011). Vascular endothelial growth factor genetic polymorphisms and haplotypes in female patients with bisphosphonate-related osteonecrosis of the jaws. J. Oral Pathol. Med..

[25] Walter C., Pabst A., Ziebart T., Klein M., Al-Nawas B. (2011). Bisphosphonates affect migration ability and cell viability of HUVEC, fibroblasts and osteoblasts in vitro. Oral Dis..

[26] Kühl S., Walter C., Acham S., Pfeffer R., Lambrecht J.T. (2012). Bisphosphonate-related osteonecrosis of the jaws—A review. Oral Oncol..

[27] Campisi G., Fedele S., Fusco V., Pizzo G., Di Fede O., Bedogni A. (2014). Epidemiology, clinical manifestations, risk reduction and treatment strategies of jaw osteonecrosis in cancer patients exposed to antiresorptive agents. Future Oncol..

[28] Pabst A.M., Ziebart T., Ackermann M., Konerding M.A., Walter C. (2014). Bisphosphonates’ antiangiogenic potency in the development of bisphosphonate-associated osteonecrosis of the jaws: Influence on microvessel sprouting in an in vivo 3D Matrigel assay. Clin. Oral Investig..

[29] Christodoulou C., Pervena A., Klouvas G., Galani E., Falagas M.E., Tsakalos G., Visvikis A., Nikolakopoulou A., Acholos V., Karapanagiotidis G. (2009). Combination of bisphosphonates and antiangiogenic factors induces osteonecrosis of the jaw more frequently than bisphosphonates alone. Oncology.

[30] Koch F.P., Walter C., Hansen T., Jäger E., Wagner W. (2011). Osteonecrosis of the jaw related to sunitinib. Oral Maxillofac. Surg..

[31] Ponzetti A., Pinta F., Spadi R., Mecca C., Fanchini L., Zanini M., Ciuffreda L., Racca P. (2015). Jaw osteonecrosis associated with aflibercept, irinotecan and fluorouracil: Attention to oral district. Tumori.

[32] Antonuzzo L., Lunghi A., Giommoni E., Brugia M., Di Costanzo F. (2016). Regorafenib Also Can Cause Osteonecrosis of the Jaw. J. Natl. Cancer Inst..

[33] Fusco V., Campisi G., Numico G., Migliorati C.A., Santini D., Bedogni A. (2016). RE: Regorafenib Also Can Cause Osteonecrosis of the Jaw. J. Natl. Cancer Inst..

[34] Hicklin D.J., Ellis L.M. (2005). Role of the vascular endothelial growth factor pathway in tumor growth and angiogenesis. J. Clin. Oncol.

[35] Renk M., Crino’ L. (2009). Advances in anti-VEGF and anti-EGFR therapy for advanced non-small cell lung cancer. Lung Cancer.

[36] Gordon C.R., Rojavin Y., Patel M., Zins J.E., Grana G., Kann B., Simons R., Atabek U. (2009). A review on bevacizumab and surgical wound healing. An important warning to all surgeons. Ann. Plast. Surg..

[37] Scappaticci F.A., Fehrenbacher L., Cartwright T., Hainsworth J.D., Heim W., Berlin J., Kabbinavar F., Novotny W., Sarkar S., Hurwitz H. (2005). Surgical wound healing complications in metastatic colorectal cancer patients treated with bevacizumab. J. Surg. Oncol..

[38] Nakagawa M., Kaneda T., Arakawa T., Morita S., Sato T., Yomada T., Hanada K., Kumegawa M., Hakeda Y. (2000). Vascular endothelial growth factor (VEGF) directly enhances osteoclastic bone resorption and survival of mature osteoclasts. FEBS Lett..

[39] Yuan F., Chen Y., Dellian M., Safabakhsh N., Ferrara N., Jain R.K. (1996). Time-dependent vascular regression and permeability changes in established human tumor xenografts induced by an anti-vascular endothelial growth factor/vascular permeability factor antibody. Proc. Natl. Acad. Sci. USA.

[40] Nicolatou-Galitis O., Razis E., Galiti D., Galitis E., Labropoulos S., Tsimpidakis A., Sgouros J., Karampeazis A., Migliorati C. (2015). Periodontaldiseaseprecedingosteonecrosis of the jaw (ONJ) in cancer patients receiving antiresorptives alone or combined with targeted therapies: Report of 5 cases and literature review. Oral Surg. Oral Med. Oral Pathol. Oral Radiol..

[41] Garant A., Des Groseilliers S., Martin L., Ferland É., Vuong T. (2011). Late anastomotic dehiscence during bevacizumab therapy for patients with colorectal cancer. Clin. Oncol. (R. Coll. Radiol.).

[42] (2011). Bevacizumab, sunitinib: Osteonecrosis of the jaw. Prescrire Int..

[43] Hompes D., Ruers T. (2011). Review: Incidence and clinical significance of bevacizumab-related non-surgical and surgical serious adverse events in metastatic colorectal cancer. Eur. J. Surg. Oncol..

[44] Ruggiero S.L., Dodson T.B., Assael L.A., Landesberg R., Marx R.E., Mehrotra B. (2009). American Association of Oral and Maxillofacial Surgeons. Position paper on bisphosphonate-related osteonecrosis of the jaws—2009 update. J. Oral Maxillofac. Surg..

[45] Bedogni A., Fedele S., Bedogni G., Scoletta M., Favia G., Colella G., Agrillo A., Bettini G., Di Fede O., Oteri G. (2014). Staging of osteonecrosis of the jaw requires computed tomography for accurate definition of the extent of bony disease. Br. J. Oral Maxillofac. Surg..

[46] Fedele S., Bedogni G., Scoletta M. (2015). Up to a quarter of patients with osteonecrosis of the jaw associated with antiresorptive agents remain undiagnosed. Br. J. Oral Maxillofac. Surg..

[47] Thomas C., Spanidis M., Engel C., Roos F.C., Frees S., Neisius A., Hampel C., Rubenwolf P., Thüroff J.W., Walter C. (2016). Bone scintigraphy predicts bisphosphonate-induced osteonecrosis of the jaw (BRONJ) in patients with metastatic castration-resistant prostate cancer (mCRPC). Clin. Oral Investig..

[48] Fusco V., Bedogni A., Addeo A., Campisi G. (2016). Definition and estimation of osteonecrosis of jaw (ONJ), and optimal duration of antiresorptive treatment in bone metastatic cancer patients: Supplementary data from the denosumab extension study?. Support. Care Cancer.

